# Effect of different doses of *Lacticaseibacillus paracasei* K56 on body fat and metabolic parameters in adult individuals with obesity: a pilot study

**DOI:** 10.1186/s12986-023-00739-y

**Published:** 2023-03-21

**Authors:** Guzailinuer Kadeer, Wanrui Fu, Yaqi He, Ying Feng, Wei-Hsein Liu, Wei-Lian Hung, Haotian Feng, Wen Zhao

**Affiliations:** 1 Department of Nutrition, Hua Dong Hospital Affiliated to Fudan University, Shanghai, China; 2 Inner Mongolia Yili Industrial Group Co., Ltd., Hohhot, China

**Keywords:** Obesity, Probiotics, Body fat, Visceral fat area, *Lacticaseibacillus paracasei* K56, Gut microbiota

## Abstract

**Background:**

Studies have shown that probiotics have an effect on reducing body fat on a strain-specific and dose–response bases. The purpose of this study was to evaluate the effect of a novel probiotic strain *Lacticaseibacillus paracasei* K56 on body fat and metabolic biomarkers in adult individuals with obesity.

**Methods:**

74 adult subjects with obesity (body mass index ≥ 30 kg/m^2^, or percent body fat > 25% for men, percent body fat > 30% for women) were randomized into 5 groups and supplemented with different doses of K56 (groups VL_K56, L_K56, H_K56, and VH_K56: K56 capsules, 2 × 10^7^ CFU/day, 2 × 10^9^ CFU/day, 2 × 10^10^ CFU/day, 2 × 10^11^ CFU/day, respectively) or placebo (group Pla: placebo capsule) for 60 days. Subjects were advised to maintain their original dietary intake and physical activity. Anthropometric measurements, body composition assessment, and metabolic parameters were measured at baseline and after 60 days of intervention.

**Results:**

The results showed that the L_K56 group had significant decreases in percent body fat (*p* = 0.004), visceral fat area (*p* = 0.0007), total body fat mass (*p* = 0.018), trunk body fat mass (*p* = 0.003), waist circumference (*p* = 0.003), glycosylated hemoglobin(*p* = 0.002) at the end of the study compared with baseline. There were non-significant reductions in Body weight and BMI in the L_K56, H_K56, VL_K56 groups, whereas increases were observed in the placebo and VH_K56 groups compared with baseline values. In addition, K56 supplementation modulated gut microbiota characteristics and diversity indices in the L-K56 group. However, mean changes in body fat mass, visceral fat area, weight, body mass index, waist circumference and hip circumference were not significantly different between groups.

**Conclusions:**

The results suggest that supplementation with different doses of *Lacticaseibacillus paracasei* K56 has certain effect on reducing body fat and glycosylated hemoglobin, especially at a dose of 10^9^ CFU/day.

*Trial registration*: clinicaltrials.gov Identifier: NCT04980599.

## Introduction

Obesity is a complex chronic disease defined as excessive or abnormal fat accumulation that adversely affects health [1]. In recent years, the increased prevalence of obesity has reached epidemic proportions, and presents a critical public health problem worldwide because of the substantial health risks associated with increased mortality from type 2 diabetes (T2D), hypertension, and cardiovascular diseases, as well as the incidence of some cancers [2]. However, safe and effective treatments for obesity are scarce and new strategies are needed to mitigate its substantial health effects.

Obesity is mostly a multifactorial disease due to obesogenic environments, psychosocial factors and genetic variants. In 2004, scientists have first reported that, gut microbiota as an important environmental factor affects energy harvest from the diet and energy storage in the host [3]. Since then, a large number of studies have explored the relationship between obesity and gut microbiota, and revealed that the changes in the gut microbial composition and function contribute to the pathophysiology of obesity [4–6] and that their modulation may aid in the prevention and treatment of this disease [7, 8].

Probiotics are defined as “live microorganisms which, when administered in adequate amounts, confer a health benefit on the host” [9]. Studies have shown potential therapeutic effects of probiotics on obesity and related metabolic disorders by influencing and maintaining the homeostasis of gut microbiota composition and function through various mechanisms of actions such as antimicrobial activity, enhancement of barrier function, immunomodulation [9, 10]. Lactic acid bacteria, specifically *lactobacillus* and *Bifidobacterium* are the most documented probiotics that appear to have beneficial effects of reducing fat mass, regulating glucolipid metabolism. Animal studies have shown that, *Lactobacillus gasseri* SBT2055 inhibits enlargement of visceral adipocytes, reduces body weight gain, improves glucose tolerance in rodents through anti-inflammatory effects and stimulation of energy expenditure [11, 12]. In another study, supplementation with *Lactobacillus plantarum* reduced fat mass and serum lipid profile concurrently with downregulation of lipogenic gene expression in the adipocytes, and modulated gut microbiota composition, resulting in reductions in the bodyweight of high fat diet (HFD) fed obese mice [13]. Similar results have been observed in other experiments in which probiotic *Bifidobacterium longum* supplemented to HFD-fed obese animals [14, 15]. In humans, supplementation with single species [16, 17] or multiple species of probiotics [18, 19] to overweight/obese subjects at various doses reduced abdominal adiposity, waist and hip circumference or improved glucolipid metabolism for varying extents. A recent meta-analysis highlighted a positive trend of probiotics supplementation in improving anthropometric measures of overweight and obese patients with associated metabolic diseases [20]. Interestingly, a strain-specific effect on body weight and metabolism of the probiotics has also been reported; Some clinical trials also suggest that the extent of anti-obesogenic effects of probiotics may depend on both the probiotic dose and viable form used [21, 22].

In a recently published animal study, a novel probiotic strain *Lacticaseibacillus paracasei* K56, isolated from the intestine of a healthy child, was treated by gavage at various doses to HFD-fed mice for 12 weeks. The results have shown that *L. paracasei* K56 significantly reduced body and fat mass and improved lipid metabolism [23]. In another animal study, administration of *Lacticaseibacillus paracasei* K56 effectively attenuated obesity parameters, such as body weight, insulin-resistance, plasma glucose and lipids; The beneficial effects may be related to the restored host gut microbiota [24]. This indicates that, K56 might be a promising probiotic strain for prevention and treatment of obesity and related metabolic disorders.

However, the beneficial effects of this novel probiotic strain have not been proved in humans, and the appropriate dosage for human administration needs to be evaluated. In this exploratory study, we aim to evaluate the metabolic effects of K56 and confirm the appropriate administration dose in humans preliminary.

## Materials and methods

### Test materials

The test materials were kindly provided by YILI industrial company Ltd. (China). The probiotic capsules contained different doses of *L. paracasei* K56 strain (very low dose: 1 × 10^7^ colony forming units/capsule, low dose: 1 × 10^9^ colony forming units/capsule, high dose: 1 × 10^10^ colony forming units/capsule, very high dose: 1 × 10^11^ colony forming units/capsule), and was standardized with maltodextrin and microcrystalline cellulose. The ingredients of the placebo capsule were similar to the probiotic capsule but without the addition of K56. The final products looked and tasted identical to each other. Participants were instructed to take two capsules per day before breakfast for 60 days.

### Study participants

The participants were recruited for the study at the Huadong hospital affiliated to Fudan University, Shanghai, China. A total of 74 subjects with obesity were initially signed informed consent. The inclusion criteria were as follows: (1) Body mass index (BMI) ≥ 30 kg/m^2^, or percentage of body fat (PBF) assessed by electrical bioimpedance ≥ 25% formen and ≥ 30% for women; (2) Age >18 and ≤ 60 years. The selected subjects were excluded from the study if they had any of the following conditions: (1) Patients with severe chronic diseases (coronary heart disease, uncontrolled diabetes, hypertension, mental disorders, cancer, hepatic or renal dysfunctions, etc.) and their complications; (2) Patients with severe allergy, gastrointestinal diseases, immunodeficiency; (3) Hyperthyroidism or hypothyroidism, Cushing syndrome, or any other disease affecting the results of the study; (4) History of administration of drugs affecting body fat or functional foods/supplements for obesity improvement in the past two months; (5) Use of any weight control measures (diet, exercise, etc.) in the past month; (6) Participation in any other clinical trials within the previous 3 months; (7) Unable to maintain their current lifestyle during the study period. (8) Failure to take the study products as required, or failure to follow up on time.

### Study design

This was a randomized, single blind, placebo controlled, pilot study and was approved by the Ethics Board Committee of Huadong Hospital (20200083), the protocol was registered at the U.S. National Institute of Health (clinicaltrials.gov Identifier: NCT04980599).

The recruitment was conducted through online enrollment questionnaires and telephone interviews, and subjects who met the inclusion criteria were scheduled for a baseline visit to assess their eligibility. Written informed consent was obtained from all eligible subjects who met the inclusion criteria and did not meet the exclusion criteria before enrollment. The subjects were then randomly assigned to one of the placebo group (Pla), very low dose K56 group (VL), low dose K56 group (L), high dose K56 group (H), and very high dose K56 group (VH) for a 60-day of intervention period. Randomization was performed using computer-generated random numbers by a statistician who had not participated in this study and group allocation was blinded to the participants.

The intervention period was lasted for 60 days, subjects were asked to take different doses of K56 or placebo capsules two capsules per day preferably before breakfast with the specific advice to maintain their previous dietary intake and physical activity, current treatments and lifestyles during the study period. At the first and second visit, investigators dispensed one bottle of test material (60capsules/bottle) to every participant, and to prevent any viability or shelf-life issues, capsules were delivered to participants in insulated bags with ice pack, and stored in refrigerator after delivery. During the intervention period, to make sure all participants to take capsules as we suggested, we made illustration about the usage of test material, and created a WeChat group in order to remind the participants to take capsules as we suggested every day. Compliance for the consumption of the test materials was assessed by counting the returned capsules at the second and the last visit. In addition, the investigators reviewed the questionnaires for missed doses submitted by the subjects every two weeks. The subjects also recorded about undesired adverse events and emergencies in the questionnaires. The Semi-quantitative food frequency questionnaire was used to monitor the changes of dietary habits and the daily walking step numbers recorded by motion recorder was used to monitor the changes of physical activity. Anthropometric measurements, body composition assessment and vital sign assessments were conducted at the day 0, day 30 and day 60 of the intervention period. Blood samples and fecal samples were collected for the biochemical and gut microbial analysis at the day 0 and day 60.

### Outcomes

The primary outcomes were changes in body fat percentage (PBF) and visceral fat area (VFA) from baseline to day 60. Secondary outcomes were changes in BMI, body weight, waist circumference, muscle mass, and metabolic parameters from the baseline to day 60.

Body weight and body compositions, including body fat mass, percent body fat, visceral fat area, regional body fat mass, skeletal muscle mass were assessed using a bioelectrical impedance analysis machines (Inbody770, Biospace, Korea) while the subject was fasting and wearing only light underwear. BMI was calculated as body weight divided by the square of the height. Waist circumference was measured directly on the skin between the lowest rib margin and the iliac crest while the subject was in a standing position using a plastic measuring tape to the nearest 0.1 cm. After 10 min of rest, blood pressure was measured in a sitting position by a trained researcher using automatic BP monitor (U16, Omron,) on the left arm.

Blood samples were collected after 10–12 h overnight fasting, and were analyzed for serum total cholesterol (TCH), high-density lipoprotein cholesterol (HDL-C), low-density lipoprotein cholesterol (LDL-C), triglycerides (TG), fasting blood glucose (FBG), insulin (INS), glycosylated hemoglobin (HbA1c), hepatic and renal functions, white blood cells by using routine laboratory methods at Hua Dong Hospital affiliated to Fudan University.

### Fecal microbiome analysis

#### Sample collection and handling

Fecal samples were collected for microbiome analysis at the baseline and after 60 days of intervention. Participants were asked to use a fecal collection box and sterile fecal container which were provided by investigators prior to collection. Samples were transported to the laboratory on ice bags, after which they were frozen and stored at − 80 °C until use. Total genomic DNA from each sample was extracted using a Hipure Soil DNA Kit (Magen, Guangzhou, China) according to the manufacturer’s instructions and quantified with a Nanodrop spectrophotometer (Thermo Scientific, Waltham, MA, USA), A260/A280 ratios were measured to confirm the purity of DNA. DNA samples were snap frozen and stored at − 20 °C till used.

#### Real-time PCR analysis

The amplification, detection and melt curve analysis of DNA were performed on an ABI7900 Sequence Detection System (Applied Biosystems). The reaction mixture (10 μl) contained 5 μl 2 × Master mix, 0.2 μl of each of the forward and reverse primers, 1 μl of ROX, 1 μl of template DNA. The amplification program consisted of 1 cycle of 95 °C for 5 min; 40 cycles of 95 °C for 15 s, 60 °C for 1 min; followed by melting curve cycling. A standard curve from genomic DNA extracted from a pure K56 strain culture was used. Each plate was run with non-template control.

#### 16S rDNA gene sequencing and bioinformatics analysis

The sequencing library was constructed using a MetaVX Library Preparation Kit (GENEWIZ, Inc., South Plainfield, NJ). Briefly, 20–30 ng of DNA was used to generate amplicons that cover V3 and V4 hypervariable regions of the 16 s rDNA gene of bacteria. The forward primer contains the sequence ‘CCTACGGRRBGCASCAGKVRVGAAT’ and the reverse primers contain the sequence ‘GGACTACNVGGGTWTCTAATCC’. The 25 μl PCR mixture was prepared with 2.5 μl of TransStart buffer, 2 μl of dNTPs, 1ul of each primer, 0.5 μl of TransStart Taq DNA polymerase and 20 ng template DNA. The PCR is performed by the following program: 3 min of denaturation at 94 °C, 24 cycles of 5 s at 95 °C, 90 s of annealing at 57 °C, 10 s of elongation at 72 °C, and a final extension at 72 °C for 5 min. Indexed adapters were added to the ends of the amplicons by limited cycle PCR. Finally, the library is purified with magnetic beads.

The concentration is detected by a microplate reader(Tecan, Infinite 200 Pro) and the fragment size is detected by 1.5% agarose gel electrophoresis which is expected at ~ 600 bp. Next generation sequencing was conducted on an Illumina Miseq Platform (Illumina, San Diego, USA). PE300 paired-end sequencing was performed according to the manufacturer’s instructions.

After sequencing, Illumina MiSeq raw data were sorted by sample using index sequences, and paired-end FASTQ files were generated for each sample. The sequencing adapter sequence and F/R primer sequence of the target gene region were removed, bases with Phred quality score lower than 20, and sequences less than 200 bp in length were removed using Cutadapt (v1.9.1, https://cutadapt.readthedocs.io/en/stable/). After sequencing, error-corrected paired-end sequences were assembled into one sequence, and sequences containing N and chimeric sequences were removed, resulting effective sequences for OUT clustering (The procedures were conducted by GENEWIZ, Inc., South Plainfield, NJ). VSEARCH (1.9.6) was used for clustering (sequence similarity is set to 97%) with reference data base Silva138. Then the representative sequences of OTUs were analyzed by RDP classifier (Ribosomal Database Program) Bayesian algorithm, and the community composition of each sample was counted at different species classification levels. Based on the obtained OTU analysis results, the α diversity information such as ACE, Shannon, Simpson and Chao1 indices were calculated to confirm the species diversity and uniformity of the microbial community in the sample using QIIME 1.9.1. Based on Bray–Curtis distance, beta diversity between samples (information about microbial community diversity between samples in comparison groups) was determined, and relationships between the samples were visualized using principal coordinate analysis (PCoA) plots. Linear discriminant analysis effect size (LEfSe) was performed using LEfSe software (v1.0, https://huttenhower.sph.harvard.edu/galaxy/).

### Statistical analysis

Prism 8.0.1 (GraphPad, San Diego, CA, USA) was used for statistical analyses on body composition and blood parameters. For continuous variables, normality tests were performed using Shapiro–Wilk tests. Normally distributed data were expressed as mean ± standard deviation and were analyzed by one-way ANOVA test with multiple comparisons by controlling the false discovery rate (Benjamini, Krieger, & Yekutieli); data with skewed distribution were expressed as median (interquartile range) and were analyzed by Kruskal–Wallis test. To test the differences between the endpoint and baseline values, the paired t-test was conducted if the data were normally distributed or Wilcoxon signed rank test if the data distribution was skewed. Significant differences in the relative abundance of microbial phyla, genera, and alpha diversity were analyzed using R software. Kruskal–Wallis test was used for between group comparisons and Wilcoxon rank-sum test for within group comparisons. A false discovery rate (FDR) based on the Benjamini–Hoch-berg (BH) adjustment was applied for multiple comparisons. A *p* value < 0.05 was considered to be significant.

## Results

### Baseline characteristics of subjects

**Fig. 1:**
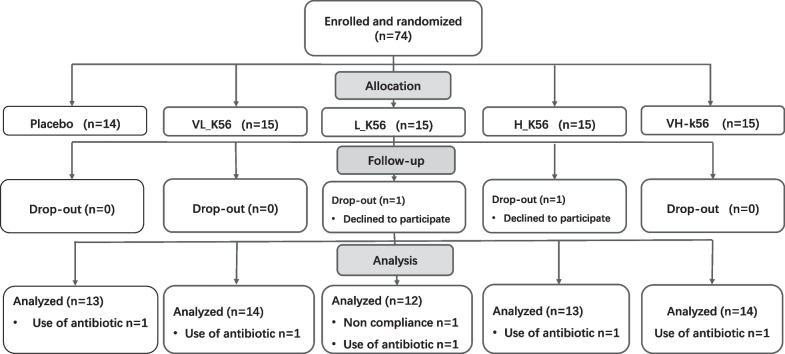
Flow diagram of enrollment, assignment, and follow-up of study participants

The study populations and reasons for exclusions are shown in Fig. 1. Seventy-four eligible subjects were enrolled in this study and randomized into five groups for 60-day intervention. Two subjects were dropped-out during the intervention period for personal reasons (one in the L_K56 group and one in the H_K56 group). A total of seventy-two subjects completed the 60-day intervention; however, one subject with < 85% treatment compliance, five subjects who administered antibiotics within two weeks before sample collection were excluded from the analysis. Therefore, a total of 66 subjects were included in the data analysis. No adverse events were reported as reasons for dropout. The baseline characteristics of the subjects who completed the study without major protocol violations are summarized in Table 1. The demographics of the subjects were similar among the different groups. There were no significant differences between groups in anthropometric variables, lipid profiles, glycosylated hemoglobin, fasting blood glucose and parameters of liver, renal functions at the baseline.

**Table 1:** Baseline characteristics of subjects

Variables	Placebo (n = 13)	VL_K56 (n = 14)	L_K56 (n = 12)	H_K56 (n = 13)	VH_K56 (n = 14)	*p* value
Gender, F/M	6/7	9/5	8/4	8/5	8/6	0.85
Height, m	166.70 ± 10.64	165.20 ± 9.28	165.30 ± 7.18	165.90 ± 10.79	163.90 ± 8.65	0.95
Age, year	40.15 ± 9.78	39.00 ± 7.74	38.42 ± 9.59	44.92 ± 8.03	39.14 ± 10.52	0.38
Weight, kg	88.84 ± 23.57	80.38 ± 17.26	86.96 ± 14.35	80.92 ± 17.28	81.34 ± 18.60	0.67
BFM, kg	34.48 ± 12.18	30.96 ± 9.73	34.34 ± 7.24	30.58 ± 6.74	31.13 ± 8.92	0.68
BMI, kg/m^2^	31.62 ± 5.70	29.27 ± 4.77	31.72 ± 4.05	29.12 ± 3.77	29.99 ± 4.71	0.45
PBF, %	38.71 ± 7.52	38.06 ± 5.62	39.42 ± 4.59	38.10 ± 5.45	38.26 ± 5.54	0.97
VFA, cm^2^	159.90 ± 49.41	146.80 ± 47.05	159.20 ± 30.52	150.00 ± 34.37	146.90 ± 38.15	0.86
WC, cm	104.20 ± 15.99	99.99 ± 14.46	102.40 ± 10.28	100.60 ± 12.54	99.22 ± 13.17	0.88
HbA1c, %	5.70 (0.90)	5.65 (0.40)	5.65 (0.60)	5.60 (0.30)	5.40 (0.20)	0.25
GA, %	12.68 ± 2.35	11.61 ± 1.43	12 ± 1.51	12.55 ± 1.27	12.11 ± 1.50	0.46
FBG, mmol/L	5.60 (0.50)	5.20 (0.30)	5.00 (1.05)	5.00 (1.10)	4.65 (0.80)	0.15
TCH, mmol/L	5.06 ± 0.82	5.27 ± 0.68	5.40 ± 0.66	4.94 ± 0.76	4.97 ± 0.71	0.43
TG, mmol/L	1.87 (1.41)	1.44 (1.30)	1.86 (0.86)	1.36 (0.64)	1.20 (0.44)	0.28
ALT, U/L	23.30 (30.80)	22.95 (23.60)	28.55 (29.75)	35.50 (21.70)	20.60 (18.40)	0.35
AST, U/L	16.10 (16.60)	18.35 (6.70)	18.85 (12.85)	24.30 (9.10)	16.55 (5.40)	0.30
Creatinine, μmol/L	79.15 ± 14.65	75.11 ± 11.09	73.97 ± 10.94	71.20 ± 11.92	75.11 ± 14.71	0.63
Urea, mmol/L	5.20 (0.80)	5.10 (0.90)	5.20 (2.20)	4.80 (1.90)	5.35 (2.10)	0.90

Data are expressed as mean ± SD or median (interquartile range). A chi-square test was performed on categorical variables. One-way ANOVA test or Kruskal–Wallis test was performed on continuous variables.

BMI, body mass index; BFM, body fat mass; PBF, percent body fat; VFA, visceral fat area; HbA1c, Glycosylated hemoglobin; GA, Glycated albumin; FBG, fasting blood glucose; TCH, total cholesterol; TG, triglycerides; ALT, Alanine transaminase; AST, Aspartate Transaminase

### Food intake and activities

Subjects were advised to maintain their original dietary pattern and activity level throughout the intervention period. According to the questionnaires feedback and recorded step counts, most of the subjects were able to maintain the required consistency of dietary and activity habits throughout the intervention period. There were no significant differences between groups in dietary intake and habitual activity at the baseline and end of study.

### Adverse events and safety parameters

The adverse events reported by participants during the intervention period included loose stools, feeling of incomplete evacuation, or flatulence, which were potentially product-related. Adverse events are summarized in Table 2. The symptoms were generally mild and of short duration, and there were no any dropouts occurred due to the adverse events. There were no significant abnormal changes in measured safety parameters: vital signs, renal and hepatic function markers (Table 3).

**Table 2:** Gastrointestinal symptoms reported by the subjects

Symptoms	placebo	VL_K56	L_K56	H_K56	VH_K56	Total
Loose stools	3 (23)	5 (36)	1 (8)	4 (31)	0 (0)	13 (20)
Flatulence	5 (38)	10 (71)	9 (75)	9 (69)	7 (0.5)	40 (60)
Feeling of incomplete evacuation	2 (15)	2 (14)	1 (8)	1 (8)	5 (36)	9 (14)

Values are expressed as number (%)

**Table 3:** Changes in biomarkers of hepatic function, renal function and vital signs

	Placebo	VL_K56	L_K56	H_K56	VH_K56	*p* value
△ALT, U/L	− 1.10 (3.70)	− 0.85 (8.30)	− 2.85 (5.30)	− 1.80 (7.00)	2.50 (7.70)	0.27
△AST, U/L	− 1.40 (5.80)	− 0.55 (3.80)	− 0.75 (6.55)	− 4.00 (2.60)	0.30 (3.20)	0.69
△Creatinine, μmol/L	− 10.02 ± 7.38	− 6.00 ± 3.96	− 9.16 ± 5.82	− 8.10 ± 3.08	− 8.41 ± 5.66	0.59
△Urea, mmol/L	− 0.10 (1.40)	0 (2.300)	− 0.25 (1.20)	− 0.10 (0.60)	− 0.10 (0.70)	0.59
△DBP, mmHg	− 1.39 ± 9.16	− 1.14 ± 10.49	1.67 ± 8.66	0.15 ± 8.08	− 1.50 ± 9.49	0.90
△SBP, mmHg	− 2.39 ± 15.40	− 1.14 ± 11.60	0.33 ± 14.49	− 4.46 ± 11.58	2.86 ± 17.71	0.74
△HR, bpm	− 2.54 ± 8.77	1.42 ± 9.64	1.00 ± 7.15	− 1.62 ± 11.15	− 5.43 ± 4.83	0.23

Data are expressed as mean ± SD or median (interquartile range). △ Changes in the mean value from baseline to 60 days. *p* value obtained from one-way ANOVA test or Kruskal–Wallis test. DBP, diastolic blood pressure; SBP, systolic blood pressure; HR, heart rate;

### Efficacy analysis

#### K56 controls body and visceral fat, reduces waist circumference

**Fig. 2:**
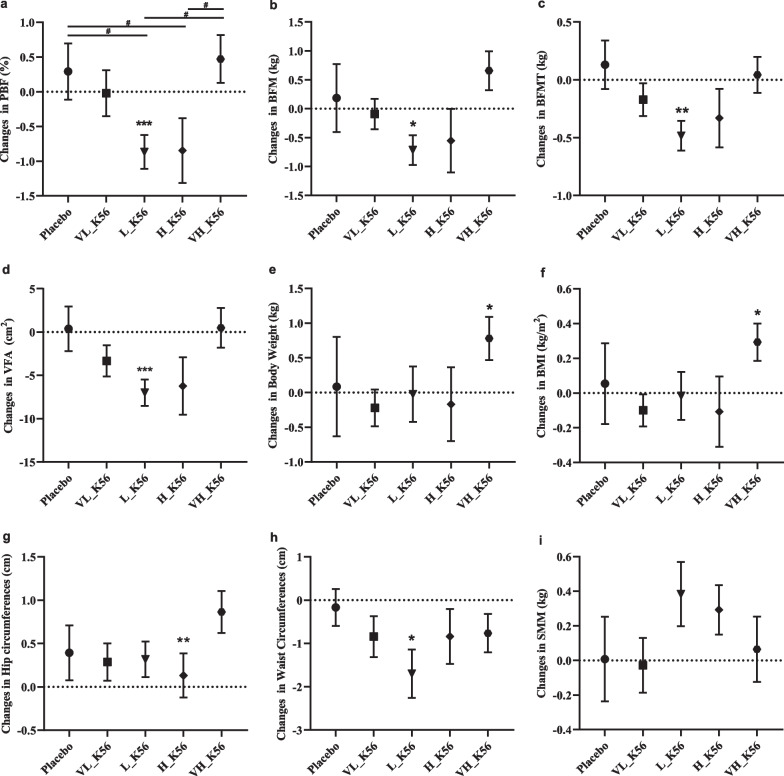
Results of anthropometric and body composition variable measurements. The graphs show **a** percent body fat, **b** body fat mass, **c** body fat mass of trunk, **d** visceral fat area, **e** weight, **f** body mass index, **g** hip circumferences, **h** waist circumference, **i** skeletal muscle mass. The data points correspond to the mean ± SEM. *p < 0.05, **p < 0.01, ***p < 0.001, comparison of baseline and 60 days values within groups (paired t-test); ^#^*p* < 0.05, ^##^*p* < 0.01, ^###^*p* < 0.001, differences in changes in mean value from baseline to 60 days between groups (one-way ANOVA)

The relative change in PBF from baseline to the end of the intervention period was the primary outcome of our study. After 60 days of probiotic intake, the mean value of PBF in L_K56 and H_K56 groups decreased compared with baseline values, especially the change in L_K56 group was statistically significant (− 0.867%, *p* = 0.004). In the placebo and VH_K56 groups, there were non-significant increases in PBF from baseline to 60 days (0.29%, 0.47%), resulting in significant differences in the mean value of changes in L_K56, H_K56 groups compared to placebo and VH_K56 groups. The total body fat mass was significantly reduced in the probiotic L_K56 group (− 0.72 kg, *p* = 0.018) at the end of the study compared with baseline. There were observations of non-significant reductions in BFM in the VL_K56, H_K56 groups, and non-significant increases in placebo and VH_K56 groups. Changes in body fat mass were most pronounced in the trunk area and a similar pattern was observed in the visceral fat area (Fig. 2). Body weight and BMI were not significantly reduced in L_K56, H_K56, VL_K56 groups, whereas there were increases in placebo and VH_K56 groups compared with baseline values, the change was statistically significant in group VH_K56. The results also indicate that, in L_K56 and H_K56 groups, there was a trend towards an increase in skeletal muscle mass. Regarding to waist and hip circumferences, the reduction in waist circumference from baseline (− 1.7 cm, *p* = 0.01) in L_K56 group and the increase in hip circumference from baseline (0.86 cm, *p* = 0.003) in VH_K56 group were statistically significant, while the changes in other groups were not significantly different. However, the mean change in BFM, VFA, weight, BMI, waist circumference and hip circumferences were not significantly different between groups.

#### K56 decreased glycosylated hemoglobin compared to baseline, but did not affect lipid profiles

Table 4 shows blood lipid profile, insulin, glycosylated hemoglobin, glycated albumin, fasting blood glucose at baseline and after intervention. Serum total cholesterol, triglyceride, HDL-Cholesterol, LDL-Cholesterol were didn’t change significantly after 60 days intervention compared with baseline in all groups, and there were no significant differences in changes from baseline to 60 days between groups. A statistically significant reduction in glycosylated hemoglobin of L_K56 group was observed at the end of intervention compared with baseline, while the changes in other groups were not significant. In addition, the glycated albumin levels in placebo and VH_K56 groups were elevated from baseline with statistically significance, and the changes in other groups were not significant. However, there were not significant differences in changes of the abovementioned variables among all groups. Insulin concentration and C-peptides were not change significantly within groups, and did not differ significantly between groups.

**Table 4:** Biochemical measurements

Variables	Placebo	VL_K56	L_K56	H_K56	VH_K56	*p* value
HbA1c, %						
Baseline	5.70 (0.90)	5.65 (0.40)	5.65 (0.60)	5.60 (0.30)	5.40 (0.20)	
60 days	5.70 (0.60)	5.55 (0.40)	5.45 (0.50)**	5.40 (0.20)	5.30 (0.20)	
Change	− 0.10 (0.40)	− 0.10 (0.10)	− 0.20 (0.20)	− 0.10 (0.20)	0.00 (0.20)	0.12
GA, %						
Baseline	11.90 (2.40)	11.95 (2.80)	12.05 (1.90)	12.50 (1.30)	11.65 (2.00)	
60 days	11.90 (2.70)*	11.95 (2.60)	12.45 (2.30)*	12.60 (1.40)	12.15 (2.30)*	
Change	0.40 (0.60)	0.30 (0.30)	0.30 (0.50)	0.10 (0.20)	0.20 (0.70)	0.69
FBG, mmol/L						
Baseline	5.60 (0.50)	5.20 (0.30)	5.00 (1.05)	5.00 (1.10)	4.65 (0.80)	
60 days	5.30 (1.10)	5.15 (0.80)	5.25 (1.15)	4.80 (1.40)	4.60 (1.00)	
Change	− 0.30 (0.80)	0.050 (0.80)	0.00 (0.40)	0.00 (0.50)	− 0.05 (0.10)	0.73
HDL-C, mmol/L						
Baseline	1.39 ± 0.33	1.48 ± 0.28	1.41 ± 0.39	1.42 ± 0.31	1.43 ± 0.35	
60 days	1.289 ± 0.33	1.47 ± 0.29	1.36 ± 0.32	1.43 ± 0.34	1.43 ± 0.31	
Change	− 0.11 ± 0.18	− 0.01 ± 0.12	− 0.05 ± 0.11	0.01 ± 0.16	− 0.002 ± 0.10	0.19
LDL-C, mmol/L						
Baseline	3.05 ± 0.86	3.28 ± 0.62	3.41 ± 0.46	3.03 ± 0.65	3.02 ± 0.61	
60 days	3.14 ± 0.79	3.62 ± 0.96	3.33 ± 0.58	3.27 ± 0.55	3.16 ± 0.65	
Change	0.09 ± 0.51	0.34 ± 0.66	− 0.08 ± 0.42	0.25 ± 0.38	0.14 ± 0.33	0.23
TCH, mmol/L						
Baseline	5.06 ± 0.82	5.27 ± 0.68	5.40 ± 0.66	4.94 ± 0.76	4.97 ± 0.71	
60 days	5.08 ± 0.93	5.45 ± 1.05	5.20 ± 0.72	5.09 ± 0.63	4.97 ± 0.75	
Change	0.02 ± 0.69	0.18 ± 0.69	− 0.20 ± 0.46	0.15 ± 0.44	− 0.00 ± 0.36	0.44
TG, mmol/L						
Baseline	1.87 (1.41)	1.44 (1.30)	1.86 (0.86)	1.36 (0.64)	1.20 (0.44)	
60 days	2.50 (1.66)	1.37 (0.81)	1.69 (1.08)	1.31 (0.65)	1.32 (0.57)	
Change	0.23 (1.05)	− 0.08 (0.26)	0.05 (0.75)	0.10 (0.33)	0.03 (0.44)	0.44
Peptide C, ng/mL						
Baseline	2.80 (1.60)	2.80 (1.10)	3.35 (2.00)	2.60 (0.90)	2.85 (1.20)	
60 days	2.70 (1.70)	3.10 (2.10)	3.00 (1.75)	2.90 (1.20)	2.80 (1.10)	
Change	0.10 (0.70)	− 0.10 (0.80)	0.00 (0.95)	0.10 (0.30)	− 0.10 (0.40)	0.70
Insulin, μU/mL						
Baseline	14.30 (7.30)	13.50 (5.90)	18.30 (20.70)	13.70 (5.00)	13.55 (10.30)	
60 days	13 (10.10)	13.70 (12.50)	16.55 (13.75)	15.10 (5.30)*	16.60 (8.50)	
Change	3.30 (8.30)	1.95 (5.10)	0.25 (6.25)	1.50 (4.20)	0.95 (5.00)	0.80

Data are expressed as mean ± SD or median (interquartile range). Differences in changes in mean values from baseline to 60 day between groups, *p* value obtained from One-way ANOVA test or Kruskal–Wallis test. *p < 0.05, **p < 0.01, obtained from paired t-test or Wilcoxon signed rank test

HbA1c, glycosylated hemoglobin; GA, glycated albumin; FBG, fasting blood glucose; HDL-c, high density lipoprotein cholesterol; LDL-c, low density lipoprotein cholesterol; TCH, total cholesterol; TG, triglycerides

### Microbiome analyses

#### 60 days supplementation with *Lacticaseibacillus paracasei* K56 increased fecal K56 levels determined by qPCR.

Table 5 shows the number of detected positive samples and average quantity of fecal K56 determined by qPCR at the baseline and after 60-day intervention. There were no significant differences in number of positive samples and average quantity of K56 between all groups at baseline. After 60-day of intervention, the number of samples with increased fecal K56 levels in all probiotic supplemented groups were significantly higher compared with the placebo group, where there were no elevated K56 levels in any subject.

**Table 5:** Quantitative analysis of K56 in feces at the baseline and day 60 by qPCR

Group	Baseline		60 days		Increased fecal K56 level^b^
	Detected positive^a^	Average quantity in positive samples [log^concentration^/ul original DNA]	Detected positive	Average quantity in positive samples [log^concentration^/ul original DNA]	
Placebo	4/14	4.331 ± 0.270	3/14	4.887 ± 0.672	0/14
VL_K56	6/15	4.411 ± 1.481	11/15	3.526 ± 1.166	9/15
L_K56	4/14	4.098 ± 1.683	14/14	2.990 ± 0.991	12/14
H_K56	4/14	4.797 ± 1.003	13/14	1.930 ± 1.333	12/14
VH_K56	7/15	4.836 ± 1.049	15/15	1.685 ± 1.356	15/15

^a^Feces samples were analyzed from all participants who had returned both baseline and after intervention samples

^b^Participants were considered have increased fecal K56 levels when the K56 concentration was at least one log higher at the end-of-study compared to baseline, while when the concentration was undetectable or remained within one log compared to baseline they were considered didn’t have increases

#### 60 days supplementation with *Lacticaseibacillus paracasei* K56 modulated gut microbial diversity and composition

**Fig. 3:**
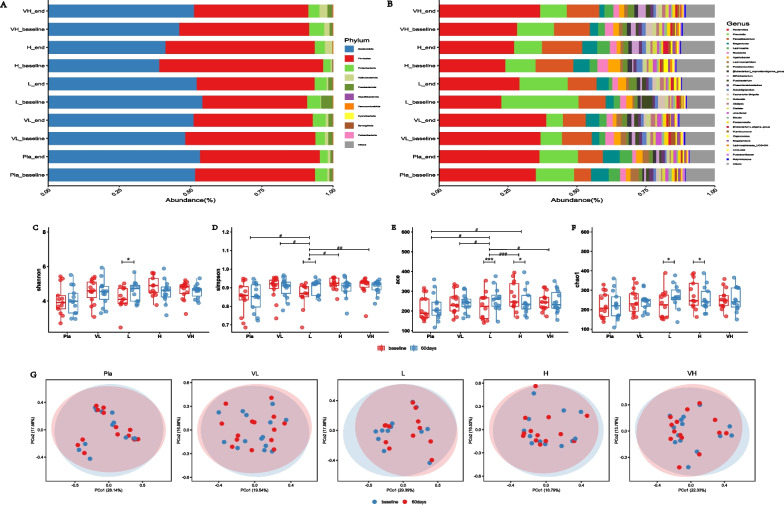
Bacterial abundance at phylum and genus level, alpha diversity, beta diversity. **A** Bacterial abundance at the phylum level at baseline and end of study in placebo and probiotic groups. **B** bacterial abundance at the genus level at baseline and end of study in placebo and probiotic groups. Boxplots show the alpha diversity of bacterial communities at baseline and after intervention in placebo and probiotic groups for **C** Shannon, **D** Simpson, **E** ACE, **F** Chao1 indices. **G** Principal coordinate analysis (PCoA) showing the microbial community distance between baseline and end of study in each group

Based on the results of the 16 rDNA sequences (V3–V4 region) using MiSeq performed in all groups, the bacterial group was dominated by the phyla *Bacteroidota*, *Firmicutes, Proteobacteria, Actinobacteriota* and *Fusobacteriota* (Fig. 3A).

A small portion of the phyla *Desulfobacterota, Verrucomicrobiota, Cyanobacteria, Synergistetes, Patescibacteria Patescibacteria* and *campilobacterota* appeared in probiotic and control groups. At baseline, bacterial phylum did not differ significantly between groups, and there were no significant changes in the abundance of bacterial phylum from baseline to end of intervention in all groups. Considering the *Firmicutes* to *Bacteroides* ratio, the H_K56 group had higher F/B ratio than other groups at the baseline, but there were no significant differences among all groups (median for Pla, VL_K56, L_k56, H_K56, VH_K56 groups were 0.65, 0.90, 0.75, 1.43, 0.99 respectively, *p* > 0.05). The changes of B/F ratio from baseline to end-of-study were not differed significantly among all groups (median of changes − 0.05, 0.01, 0.17, − 0.20, − 0.17 for Pla, VL_K56, L_K56, H_K56, VH_K56 respectively).

At the genus level (Fig. 3B), the abundance of genus *Bacteroides* at baseline was lower and the abundance of genus *Prevotella* was higher in L_K56 group than other groups, but they didn’t differ significantly between groups. After 60 days of intervention, the abundance of genus *Parabacteroides* in L_K56 group increased significantly from baseline (*p* = 0.01, Wilcoxon test). The genus *Bacteroides, Alistipes, Parasutterella* in L_K56 group experienced increases in abundance and genus *Prevotella* in L_K56 group experienced decrease in abundance through the intervention period. The genus *Bacteroides* was increased slightly in VH_K56 group. *Agathobacter* in H_k56 group was decreased significantly (*p* = 0.035).

Alpha diversity indices (Fig. 3C–F), including ACE index, Chao1 index, Shannon index and Simpson index, indicate the richness and evenness of gut microbial community. At the end of the intervention, there were significant increases in ACE, Shannon, Chao1 and Simpson indices from baseline in the L_K56 group; And significant decreases in ACE and Chao1 indices in the H_K56 group. The increases in ACE and Shannon indices in the L_K56 group were differed significantly from other groups. The β diversity of each group was examined by principal coordinate analysis (PCoA), the results showed that there were no significant differences from baseline to end of study in each group except for a slight separation in L_K56 group (Fig. 3G).

**Fig. 4:**
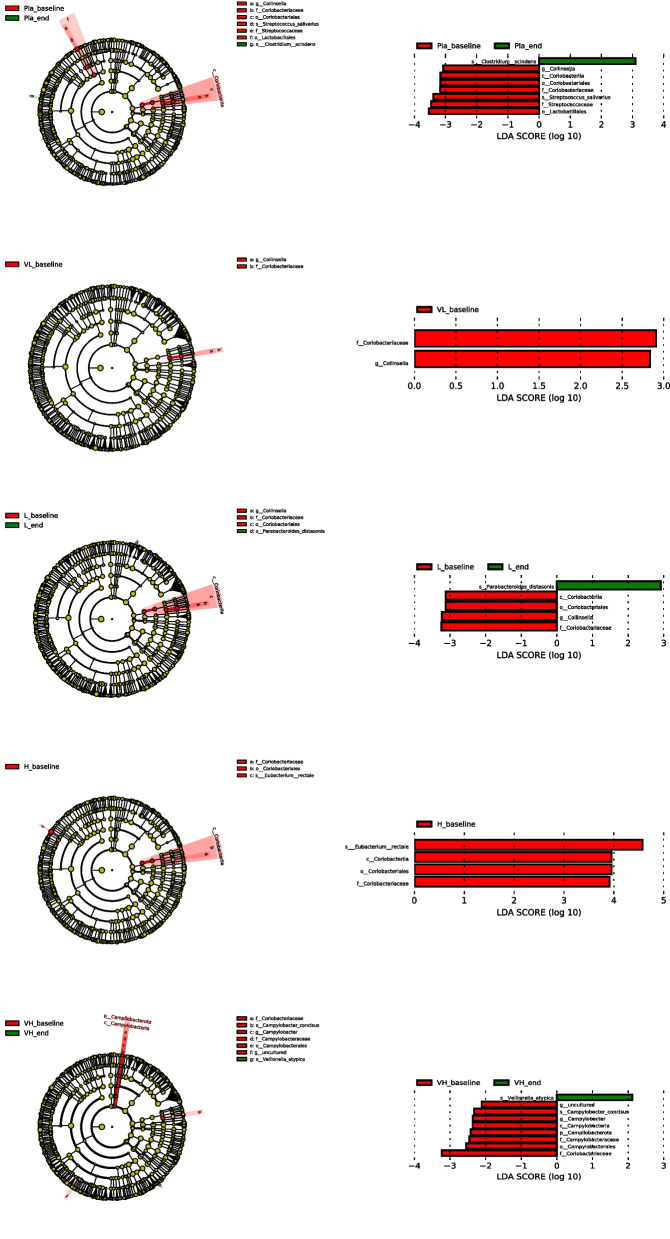
Linear discriminant analysis (LDA) effect size (LEfSe) was used to calculate the taxa that best discriminated between the baseline and end of study in each group. Taxa that reached a linear discriminant analysis score (log10) > 2.0 are highlighted and labelled at taxonomic levels from phylum to species

The differentially abundant taxa between baseline and end of study in each group was identified by Linear discriminant analysis Effect Size (Fig. 4). At phylum level, there were no significantly differentiated bacteria among top ten phyla in all groups. The abundance of class *Coriobacteria* and its members (order *Coriobacterials*, family *Coriobacteriaceae*, genus *Collinsella*) decreased significantly at the end of study compared to baseline in each group. The abundance of *Parabacteroids distasonis* in L_K56 group, *Clostridium scindens* in placebo group and *Veillonella atypica* in VH_K56 group were significantly increased than baseline.

## Discussion

This study presents a comprehensive analysis of the effects of supplementation with a novel probiotic strain on obesity, metabolic parameters and gut microbiota in free-living adults with obesity. After 60 days of intervention period, we found that supplementation with *Lacticaseibacillus paracasei* K56 at a daily dose of 10^9^ CFU determined beneficial effects on obesity and glucose metabolism by reducing body fat mass, body fat percentage, trunk fat mass and visceral fat area, waist circumferences and glycosylated hemoglobin. Since central fat has a greater negative impact on the metabolic risk associated with obesity, the reduction of fat compartment may represent a beneficial effect of probiotics, even though there was no significant reduction in body weight. However, the effects of K56 were no longer significant when the dose of supplementation was higher (10^10^ CFU/day) or lower (10^7^ CFU/day), and at very high doses(10^11^ CFU/day), there was a trend toward opposite effects. Such dose–response effects of probiotics have been observed in a previous clinical study in which, after obese adults were randomized to receive low-dose *lactobacillus gasseri* BNR17 (BNR-L, 10^9^ CFU/day), or high-dose BNR17 (BNR-H, 10^10^ CFU/day) for 12 weeks, reduced visceral adipose tissue was only observed in high dose of *L. gasseri* BNR17 group [21]. In a randomized clinical trial, obese subjects received 200 g/d fermented milk contains 10^8^ CFU/g *Lactobacillus gasseri* SBT2055 for 12 weeks, the abdominal visceral and subcutaneous fat area reduced from baseline significantly by an average of 4.6% and 3.3% respectively [16]. However, when the concentrations of SBT2055 were 10^6^ or 10^7^ CFU/g, significant reductions were didn’t present, suggesting a possible diminution of effectiveness at lower doses [17]. However, in a recent study, researchers observed that there were no unequivocal relationships between the effect of probiotics and the dose [25]. In a previously reported animal study, high fat diet induced obese mice were treated by gavage five times a week with freshly prepared K56 (10^6^ CFU/day or 10^8^ CFU/day) alone or in combination with α- galactooligosaccharides for 12 weeks. After treatment, all probiotic groups significantly decreased body weight gain and visceral fat than high fat diet (HFD) group, especially at the dosage of 10^8^CUF/day alone or combined with α-GOS had lower body weight and fat gain than 10^6^ CFU/day group [23]. In another animal study, HFD-fed mice were administered K56 suspension of 10^7^ CFU/day, 10^9^ CFU/day, and 10^11^ CFU/day. After 10 weeks of intervention, the three K56 groups did lowered the weight gain and abdominal fat than HFD group, and there were no significant differences between the three k56 groups. However, the abdominal fat by MRI scanning in 10^7^group was significantly higher than normal diet (ND) group, whereas no significant increases or comparable to ND group in 10^9^ and 10^11^ groups. Moreover, regarding the impact to glucose metabolism, the AUC of oral glucose tolerance test was significantly reduced in 10^9^ and 10^11^ groups than HFD group, especially in the 10^9^group [24]. *Lactobacillus johnsonni* 3121 and *Lactobacillus. rhamnosus* 86 were also evaluated for their anti-obesity effects using a high-fat diet-induced obese mouse model. Daily oral administration of *L. johnsonni* 3121 and *L. rhamnosus* 86 for 12 weeks (10^10^ CFU/day) significantly improved serum lipid profile and downregulated the expression of genes related to adipogenesis and lipogenesis in epididymal white adipose tissue of high-fat diet fed obese mice (*p* < 0.05) [26]. Fat mass expansion of ketonic diet induced diabetic mice was ameliorated by treatment with *Bifidobacterium animalis* ssp. *Lactis* 420 at a dose of 10^10^ CFU/day (*p* = 0.020), and there was a marked trend of fat mass reduction by 10^9^ CFU/day (*p* = 0.066) [27]. These findings suggest that, the dose probiotics need varies greatly depending on the strain. Although the recommended intake of probiotics is mainly between 10^7^ and 10^11^ CFU/day, some strains have been shown to be efficacious at lower levels, while some requires substantially more [28]. In this study, obesity parameters such as PBF, VFA, BFM, WC were reduced significantly after treatment with K56 in L_K56 group, and trend to improvements were observed in VL_K56 and H_K56 groups. Although the results were not as robust as animal experiments, but generally consistent with the results of above-mentioned animal experiments in which the anti-obesity effects of K56 were evaluated.

An accumulating body of evidence has suggested that the gut microbiota of obese individuals is characterized by a decrease of α diversity, an alteration of β diversity, an increased abundance of phylum *Firmicutes* and *Firmicutes-to-Bacteroidetes* ratio, while some other studies have suggest that no significant difference existed in obese and lean individuals concerning *Firmicutes/Bacteroidetes* ratio and the abundance of *Bacteroidetes* [29]. Numerous mechanisms of action for probiotic-mediated weight loss have been proposed. These include the modification of the gut microbiota, reduction of intestinal permeability, and modulation of the immune system [10, 30]. In our study, concerning the changes in abundance of bacterial phyla after intervention period, there were no statistically significant changes in each group. This is in accordance with a previously reported clinical trial in which multi-species probiotic includes nine strains of *Bifidobacterium* and *Lactobacillus* altered the influence of microbiota on biochemical, physiological and immunological parameters, but it didn’t affect overall composition of gut microbiota after 12-weeks administration to obese, postmenopausal women. It is noteworthy that, low-dose K56 supplementation increased the abundance of genus *Parabacteroides* and species *Parabacteroides distasonis* significantly. According to previous papers, the gut microbial community of obese patients exhibited a significant decrease in the relative abundance of several *Bacteroidetes* taxa including *Parabacteroides spp.*, *Bacteroides spp.* when compared to normal weight subjects and negatively correlated with body fat and waist circumferences [31, 32]. Besides, researchers recently have found that *Parabacteroides distasonis* could affect the proportion of secondary non-12α-hydroxylated bile acids and metabolism of glucose and lipid, ameliorate weight regain via increased thermogenesis [33]. Although bile acids were not analyzed in this study, it is possible that treatment with K56 induces weight loss in subjects with obesity by increasing the abundance of *Parabacteroides distasonis* species, followed by increased secondary non-12α-hydroxylated bile acids and increased thermogenesis. In addition, genus *Bacteroides* in L_K56 and VH_K56 groups, *Alistipes* and *Parasutterella* in L_K56 group each trended towards increased abundance in the gut after intervention. In an animal study, it has been reported that *Bacteroides* has protective effects against weight gain [34]. *Alistipes,* a genus belongs to *Bacteroidetes* phylum, has been reported to inversely correlated to adiposity, lipid, and glucose homeostasis parameters [35], and may have protective effects against some diseases, including liver fibrosis, colitis, cancer immunotherapy, and cardiovascular disease [36]. *Parasutterella* was reported to have potential role in bile acid maintenance and cholesterol metabolism [37]. After administration of K56, we also noted a trend of reduction in the abundance of *Prevotella* in L_K56 group. In a previous clinical trial, it has been reported that high abundance of *Prevotellaceae* and *Veillonellaceae* associated with obesity and impaired glucose metabolism [38]. Recently, researchers have proposed that high abundance of *Prevotella*, especially *P. copri* in the gut may be associated with excessive energy uptake and increase fat accumulation [39]. In addition, Individuals with reduced microbial gene richness present more pronounced dys-metabolism and low-grade inflammation that were the main characteristics of obesity, suggesting that reduced gut microbial diversity accompanied changes in key species is the decisive factor in obesity [40]. According to the ACE, Shannon, Simpson and Chao1 indices, there were significant changes in alpha diversity of the intestinal microbial community in L_k56 and H_K56 groups from baseline to end of study and the changes in alpha diversity in L_K56 group differed significantly from other groups. This in agreement with a previous RCT that also reported significant differences in alpha diversity after supplementation with probiotic *Lactobacillus curvatus* HY7601 and *Lactobacillus plantarum* KY1032 [41]. However, the PCoA scatter plot for baseline and after intervention didn’t differed in each group except for a slight trend to separation in L_K56 group. Taken together, the results indicate that K56 administration is expected to enrich the microbial community, modulate the gut microbiota associated with obesity. This is in consistent with previous preclinical study in which K56 supplementation restored the gut microbiota of HFD fed mouse and ameliorated HFD induced obesity and associated metabolic parameters such as blood glucose and lipid profile [24]. But we didn’t observe significant changes in plasma lipid profile and fasting blood glucose in present study, except for a statistically significant reduction in HbA1c in L_K56 group. However, the average levels of plasma lipid and glucose were within normal range at baseline, and after 60 days of K56 supplementation in all groups. This result warrants further investigations in patients with hyperlipidemia and prediabetes to evaluate a metabolic benefit of K56. Also, this exploratory study enrolled a small number of individuals, which affects statistical power, especially when the effects of an intervention on clinical features were investigated. As a result, the study was not powered to deliver definitive conclusions on the end points related to energy balance. However, all the groups were randomized and investigated blindly. We may argue that any confounding factors were probably equally distributed between different groups. And we didn’t observe any improvements in Placebo group over the intervention period. Based on this exploratory study and preclinical animal studies, we could suggest that administration of K56 in adequate amount, may help improve obesity and related metabolic parameters, and the dosage as high as 10^11^ CFU/day is safe. If we take efficiency and economy into account, the dose of 10^9^ CFU/day could probably be a better option. Meanwhile, this study was a promising start for future clinical trials with propriate design to confirm and extend our study results.

## Conclusion

This was the first randomized single-blind placebo controlled exploratory study to investigate the effects of supplementation with a novel probiotic strain K56 in obese free-living adults. The results suggest that, under the condition of maintaining original dietary intake and physical activity, supplementation with different doses of *Lacticaseibacillus paracasei* K56 has certain effect on reducing body fat, improving glucose metabolism and modulating the gut microbiota to favor anti-obesity characteristics, especially at a dose of 10^9^ CFU/day.

## Data Availability

The data that support the findings of this study are available from the corresponding author Ying Feng, upon reasonable request.

## Abbreviations

BMI: Body mass index
BFM: Body fat mass
PBF: Percent body fat
VFA: Visceral fat area
HbA1: Glycosylated hemoglobin
GA: Glycated albumin
FBG: Fasting blood glucose
TCH: Total cholesterol
TG: Triglycerides
ALT: Alanine transaminase
AST: Aspartate transaminase
DBP: Diastolic blood pressure
SBP: Systolic blood pressure
ANOVA: Analysis of variance
